# Antibiotics as first-line alternative to appendicectomy in adult appendicitis: 90-day follow-up from a prospective, multicentre cohort study

**DOI:** 10.1093/bjs/znab287

**Published:** 2021-09-03

**Authors:** H Javanmard-Emamghissi, M Hollyman, H Boyd-Carson, B Doleman, A Adiamah, J N Lund, S Moler-Zapata, R Grieve, S J Moug, G M Tierney

**Affiliations:** 1 Faculty of Medicine and Health Sciences, University of Nottingham at Derby, Royal Derby Hospital, Derby, UK; 2 Upper Gastrointestinal Surgery Department, Musgrove Park Hospital, Taunton, UK; 3 Colorectal Department, Royal Derby Hospital, Derby, UK; 4 Department of Gastrointestinal Surgery, NIHR Nottingham Digestive Disease Biomedical Research Centre, Nottingham University Hospitals NHS Trust, Nottingham, UK; 5 Department of Health Services Research and Policy, London School of Hygiene and Tropical Medicine, London, UK; 6 Colorectal Department, Royal Alexandra Hospital, Paisley, UK

## Abstract

**Background:**

Uncomplicated acute appendicitis can be managed with non-operative (antibiotic) treatment, but laparoscopic appendicectomy remains the first-line management in the UK. During the COVID-19 pandemic the practice altered, with more patients offered antibiotics as treatment. A large-scale observational study was designed comparing operative and non-operative management of appendicitis. The aim of this study was to evaluate 90-day follow-up.

**Methods:**

A prospective, cohort study at 97 sites in the UK and Republic of Ireland included adult patients with a clinical or radiological diagnosis of appendicitis that either had surgery or non-operative management. Propensity score matching was conducted using age, sex, BMI, frailty, co-morbidity, Adult Appendicitis Score and C-reactive protein. Outcomes were 90-day treatment failure in the non-operative group, and in the matched groups 30-day complications, length of hospital stay (LOS) and total healthcare costs associated with each treatment.

**Results:**

A total of 3420 patients were recorded: 1402 (41 per cent) had initial antibiotic management and 2018 (59 per cent) had appendicectomy. At 90-day follow-up, antibiotics were successful in 80 per cent (1116) of cases. After propensity score matching (2444 patients), fewer overall complications (OR 0.36 (95 per cent c.i. 0.26 to 0.50)) and a shorter median LOS (2.5 *versus* 3 days, *P* < 0.001) were noted in the antibiotic management group. Accounting for interval appendicectomy rates, the mean total cost was €1034 lower per patient managed without surgery.

**Conclusion:**

This study found that antibiotics is an alternative first-line treatment for adult acute appendicitis and can lead to cost reductions.

## Teaser

Adult appendicitis is the most common emergency general surgery condition worldwide, and is normally treated with laparoscopic appendicectomy despite evidence that it can be treated safely with antibiotics. The authors followed up patients who received operative treatment and non-operative treatment with antibiotics during the first wave of the COVID-19 pandemic. Non-operative management was associated with a shorter length of hospital stay, fewer complications and a significant health cost reduction compared with operative management.

## Introduction

Antibiotics as the first-line treatment for adult acute appendicitis is an accepted, but often overlooked, strategy^1^. Recent trial evidence has reported that antibiotics can be successful and avoid surgery in the majority of uncomplicated acute appendicitis, both in the short and longer term^2–8^. In addition, international guidelines state antibiotics are safe and an effective alternative to surgery in patients with uncomplicated acute appendicitis where imaging has not shown the presence of a faecolith^9^. Despite this, implementation of antibiotics as first-line treatment has not occurred and laparoscopic appendicectomy remains the first-line treatment for acute appendicitis in Europe and the USA^9^^,^^10^.

The first wave of the SARS-COV-2 (COVID-19) pandemic raised significant concerns around the choice of surgical intervention: potentially increased morbidity and mortality in those infected with COVID-19 having an operation; and viral transmission via aerosolization during laparoscopy. These concerns led to professional surgical societies recommending non-operative management with antibiotics over surgery as first-line management of acute appendicitis^11^^,^^12^. Anticipating a significant shift in practice, the authors designed and implemented a pragmatic, observational, unselected cohort study of management of uncomplicated acute appendicitis.

The collaborative’s previous publication on the first 500 adult patients confirmed a significant shift in practice towards non-operative management of acute appendicitis and a higher number of CT scans (71 per cent) performed to aid diagnosis during the pandemic^13^. This publication also reported on the successful short-term safety and efficacy of non-operative management as first-line treatment up to 30 days from diagnosis^13^. The authors now report on the complete study cohort with the primary aim of documenting the 90-day success rate of non-operative management. Secondary aims were analysis of factors influencing outcome and reporting the relative costs of non-operative *versus* operative management at 90 days.

## Methods

Ethical approval was not required as the study collected routine, anonymized data and no clinical care was influenced.

### Study design

A prospective, multicentre study in the UK and the Republic of Ireland on patients aged at least 18 years diagnosed either clinically and/or radiologically with acute appendicitis in a secondary care setting was carried out. The patients were managed initially either with antibiotic (non-operative) or operative management. Data was collected from patients presenting from the date of the UK Government COVID-19 lockdown on 23 March 2020 (28 March 2020 in the Republic of Ireland) until 23 June 2020. Study registration was overseen by the local principal investigator at each site as either a clinical audit or service evaluation. This observational study was reported according to STROBE guidelines for observational studies, where appropriate^14^.

### Site recruitment and data collection

Any hospital in the UK or Republic of Ireland providing emergency care for patients diagnosed with acute appendicitis could participate. The protocol was published^15^, and trainee-led research collaboratives and social media (@covidharem) aided recruitment of sites. Publicity for the project was supported by The Association of Surgeons of Great Britain and Ireland (ASGBI) and the Royal College of Surgeons of England.

Local teams screened patients presenting with abdominal pain to identify patients eligible for inclusion. Once screened, collaborators entered anonymized data of patients meeting inclusion criteria to Research Electronic Data Capture (REDCap, www.project-redcap.org)^16^. The database was developed, maintained and hosted by the Major Trauma Team at Nottingham University Hospitals, UK (@EastMidsMTC).

### Group allocation

Decision for first-line/initial treatment of acute appendicitis was at the discretion of the clinical team independent of the study team. No guidance was offered by the study team although national professional bodies had issued guidance on the investigation and management of appendicitis during the pandemic^11^^,^^12^. If the decision was surgery within the first 2 days of presentation, this patient was entered into the study in the operative group; if the decision was for antibiotics, this patient entered the non-operative management group.

### Outcome measures

Admission variables were recorded for all patients including demographic data, frailty, co-morbidities, patient observations, duration of symptoms and blood inflammatory markers. Sites were asked to calculate an Adult Appendicitis Score (AAS) for each patient using an online calculator (www.appendicitisscore.com).

Patients in both cohorts were followed up for 90 days from presentation for total length of hospital stay (LOS), death, rate of hospital reattendance, operations, interventional radiology (IR) drain placement, and admission to critical care (level 2 (high dependency unit) or 3 (intensive care unit)), 30-day complication rate and total costs. Operative details were recorded including surgical approach, duration of operation, procedure, surgical findings and histology.

Patient demographics and outcomes were analysed by intention to treat by initial operative or non-operative management. Failure of non-operative management was recorded as management changing from antibiotics to surgery at least 2 days after initial assessment. Patients requiring IR drainage on initial presentation were excluded from the main analysis as initial management was neither non-operative nor operative.

### Data validation

Random number generation was used to select 15 per cent of patients entered into the database for data validation. Sites were asked to nominate an independent team member to collect data for a predetermined 25 per cent of data points for these randomly selected patients. These data were analysed centrally against the database and generated an overall data validation percentage of 98.1 per cent. Sites with data validation below 95 per cent were excluded from the analysis.

### Statistical analysis

Due to missing data, all outcome proportions were reported as the number of events/total patients with data. Descriptive data were reported as median (i.q.r.) or rates as appropriate. Differences in demographics between the non-operative and operative groups were analysed using Mann–Whitney *U* and Fisher’s exact test as appropriate. Standardized differences (SD) are presented to show the differences between groups in baseline characteristics, with values of greater than 0.1 regarded as demonstrating imbalance between the groups^17^.

In order to reduce the effects of selection bias in this non-randomized cohort, propensity score matching was performed using a probit model for the following variables: age, sex, frailty status, Adult Appendicitis Score^10^, presence of obesity, diabetes, chronic obstructive pulmonary disease (COPD), previous myocardial infarction and C-reactive protein (CRP). Matching was performed one to one to the nearest neighbour (without replacement) within a calliper of 0.1^18^. Violin plots for propensity score distributions can be found in Supplementary material online, *Figs S1* and *S2*. Following matching, conditional logistic regression was performed and differences between matched groups are presented as odds ratios and 95 per cent confidence intervals. Length of stay was analysed using negative binomial regression due to overdispersion. This outcome is presented as an incident rate ratio and 95 per cent confidence intervals. All statistical analysis was performed using Stata® version 16.1 (StataCorp, www.stata.com). The user-written commands psmatch2 and stddiff were used.

### Cost analysis

The cost analysis undertook a time horizon of 90 days, and was undertaken from the hospital perspective. The costs included were those pertaining to the index hospital admission, and any subsequent readmissions. Resource use categories were defined a priori and include those where differences between groups are likely to drive incremental costs. These are the duration of hospital admission including days in critical care and ward, duration and choice of surgical approach (open or laparoscopic), the use of antibiotics, the use of imaging and any subsequent readmissions. Total costs at 90 days were calculated by combining resource-use data at the patient level with unit costs at 2018–2019 prices in pounds sterling, reported in Euros at a conversion rate of 1:1.16. The results were subjected to extensive sensitivity analyses, including the potential for unmeasured confounding and the approach taken in the unit costs analysis. A detailed breakdown of the costings used are available in Supplementary material online, *Tables S2* and *S3*.

## Results

**Fig. 1:**
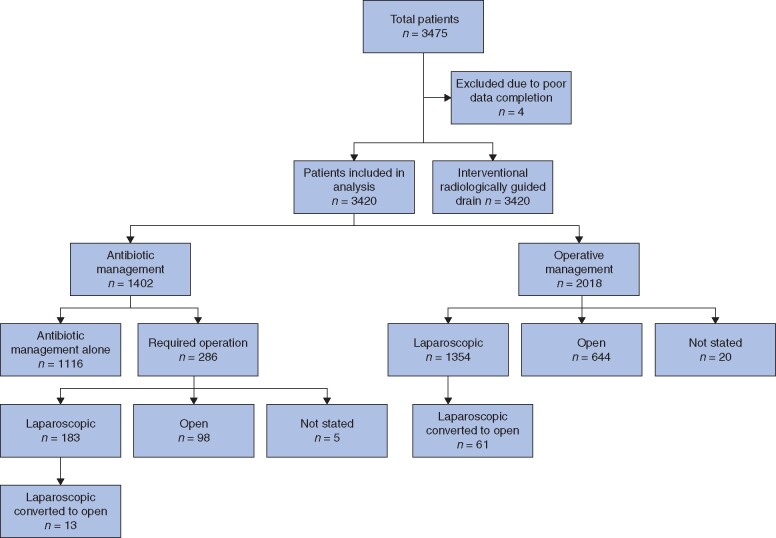
Study flow chart

A total of 3420 patients were included in the analysis (*Fig. 1*): 48 per cent (1643 patients) were female; median age 36 (i.q.r. 26–52) years. Overall COVID-19 positivity was low at 1 per cent (32 patients).

Acute appendicitis was treated first line with non-operative management in 41 per cent (1402 patients) and operative management in 59 per cent (2018 patients). Laparoscopic appendicectomy was performed in 65 per cent (1298 patients), laparoscopic converted to open in 3 per cent (61 patients) and open appendicectomy in 32 per cent (644 patients). At 90 days’ follow-up, 80 per cent (1116 of 1402) of the non-operative group had avoided operative intervention. Baseline characteristics by group are shown in *Table 1*.

**Table 1:** Demographics of included patients

Event	Total (*n* = 3420)	Operative management (*n* = 2018)	Non-operative management (*n* = 1402)	Standardized difference	***P*** †
Age (years)*	36 (26–52)	36.5 (27–52)	35 (26–52)	0.002	0.345
**Female**	1643 of 3419 (48)	919 of 2018 (46)	724 of 1401 (52)	0.13	**<0.001**
**BMI (kg/m^2^)**					
<20.0	199 of 3297 (6)	102 of 1937 (5)	97 of 1360 (7)	0.11	**0.032**
20.1–25.0	1256 of 3297 (38)	739 of 1937 (38)	517 of 1360 (38)		
25.1–30.0	1144 of 3297 (35)	665 of 1937 (34)	479 of 1360 (35)		
30.1–35.0	475 of 3297 (14)	304 of 1937 (16)	171 of 1360 (13)		
>35.1	223 of 3297 (7)	127 of 1937 (7)	96 of 1360 (7)		
**Rockwood clinical frailty score**					
Not frail (1–4)	3341 of 3419 (98)	1987 of 2018 (98)	1354 of 1401 (97)	0.12	**0.001**
Frail (5–9)	78 of 3419 (2)	31 of 2018 (2)	47 of 1401 (3)		
**Co-morbidity**					
COPD	76 of 3420 (2)	43 of 2018 (2)	33 of 1402 (2)	0.02	0.724
Myocardial infarction	96 of 3420 (3)	47 of 2018 (2)	49 of 1402 (4)	0.07	**0.046**
Diabetes	134 of 3420 (4)	76 of 2018 (4)	58 of 1402 (4)	0.02	0.592
Active cancer	34 of 3420 (1)	17 of 2018 (1)	17 of 1402 (1)	0.04	0.297
Immunosuppressed	57 of 3419 (2)	31 of 2018 (2)	26 of 1401 (2)	0.02	0.499
**Smoking status**					
Current	499 of 3411 (15)	298 of 2014 (15)	201 of 1397 (14)	0.05	0.371
Previous	283 of 3411 (8)	156 of 2014 (8)	127 of 1397 (9)		
Never	2629 of 3411 (77)	1560 of 2014 (77)	1069 of 1397 (77)		
**Adult Appendicitis Score group**					
Low risk	628 of 3420 (18)	266 of 2018 (13)	362 of 1402 (26)	0.42	**<0.001**
Intermediate risk	1765 of 3420 (52)	1016 of 2018 (50)	749 of 1402 (53)		
High risk	1207 of 3420 (30)	736 of 2018 (36)	291 of 1402 (21)		
**Duration of symptoms**					
<24 hours	1074 of 3414 (31)	653 of 2014 (32)	421 of 1400 (30)	0.15	**0.001**
25–48 hours	1155 of 3414 (34)	690 of 2014 (34)	465 of 1400 (33)		
49–72 hours	451 of 3414 (13)	273 of 2014 (14)	178 of 1400 (13)		
73–96 hours	298 of 3414 (9)	182 of 2014 (9)	116 of 1400 (8)		
>97 hours	436 of 3414 (13)	216 of 2014 (11)	220 of 1400 (16)		
**Temperature on admission**					
<37.4	2410 of 3408 (71)	1360 of 2011 (68)	1050 of 1397 (75)	0.19	**<0.001**
37.5–37.9	598 of 3408 (18)	371 of 2011 (18)	227 of 1397 (16)		
38–38.4	274 of 3408 (8)	189 of 2011 (9)	85 of 1397 (6)		
>38.5	126 of 3408 (4)	91 of 2011 (5)	35 of 1397 (3)		
**Heart rate on admission**					
>90 beats per minute	1330 of 3406 (39)	825 of 2008 (41)	505 of 1398 (36)	0.1	**0.004**
**Imaging**					
Ultrasound	610 of 3420 (18)	293 of 2018 (15)	317 of 1402 (23)	0.21	**<0.001**
Magnetic resonance imaging	32 of 3420 (1)	17 of 2018 (1)	15 of 1402 (1)	0.02	0.589
Computed tomography	2402 of 3420 (70)	1418 of 2018 (70)	984 of 1402 (70)	0.002	0.970
Ultrasound and CT	150 of 3420 (4)	68 of 2018 (3)	82 of 1402 (6)	0.12	**0.001**
					
**Appendix histology**			Unsuccessful NOM (*n* = 286)		
Acute appendicitis		1855 of 1974 (94)	232 of 277 (84)	0.31	**<0.001**
Chronic appendicitis		10 of 1974 (0.5)	7 of 277 (3)	0.17	**0.003**
Malignancy		29 of 1974 (1)	4 of 277 (1)	0.002	1.000
Neuroendocrine tumour		7 of 1974 (0.4)	3 of 277 (1)	0.09	0.115
Normal		55 of 1974 (3)	18 of 277 (6)	0.17	**0.003**
Other		18 of 1974 (1)	9 of 277 (3)	0.16	**0.004**
**Operative approach**					
Open		644 of 1998 (32)	98 of 281 (35)	0.06	0.378
Laparoscopic		1293 of 1998 (65)	170 of 281 (60)	0.09	0.184
Laparoscopic converted to open		61 of 1998 (3)	13 of 281 (5)	0.06	0.205
**Operation performed**					
Appendicectomy		1972 of 1999 (99)	266 of 281 (95)	0.21	**<0.001**
Right hemicolectomy		22 of 1999 (1)	10 of 281 (4)	0.16	**0.004**
Laparoscopy/washout		10 of 1999 (0.5)	4 of 281 (2)	0.10	0.083
Other		2 of 1999 (0.1)	1 of 281 (0.4)	0.05	0.326
**Grade of surgeon**					
Consultant		414 of 2012 (21)	83 of 284 (29)	0.23	**0.003**
Registrar		1280 of 2012 (64)	153 of 284 (54)		
Core trainee		239 of 2012 (12)	32 of 284 (11)		
Other		79 of 2012 (4)	16 of 284 (6)		

Values in parentheses are percentages unless indicated otherwise; bold text denotes values of statistical significance;

*values are median (i.q.r.).

† *P* values of significance are highlighted in bold. NOM, non-operative management

Patients with laboratory-confirmed COVID-19 infection on admission were more likely to undergo non-operative management: 11 patients in the operative group and 21 in the non-operative management group (*P* < 0.001). Blood inflammatory markers were significantly different between the two groups; the median CRP on admission of those in the operative group was 49 mg/L (i.q.r. 14–127) and in the non-operative group was 33 mg/L (i.q.r. 8–98) (*P* < 0.001), and white cell count was 13.9 10^9^/L (i.q.r. 11.1–16.8) and 12.5 10^9^/L (i.q.r. 9.8–15.6) respectively (*P* < 0.001).

Patients who had non-operative management were more likely to have an ultrasound scan performed than those in the operative group (23 *versus* 15 per cent, *P* < 0.001). CT was performed in 70 per cent of patients in both groups, with a faecolith present in 30 per cent (713 patients) of all patients. Of those found to have a faecolith, 73 per cent (523 patients) were managed operatively and 27 per cent (190 patients) were initially managed non-operatively (*P* < 0.001).

### Outcomes after unsuccessful non-operative management

Non-operative management was unsuccessful leading to surgery in 20 per cent (286) of patients; 60 per cent (170 patients) had a laparoscopic approach, 35 per cent (98 patients) open and 5 per cent (13 patients) had a laparoscopic converted to open procedure (*Fig. 1*). The median time interval to change of management from non-operative to operative management was 2 (i.q.r. 2–7) days. The majority of decisions to change management strategy occurred in the first week (76 per cent), with 80 per cent occurring in the first 2 weeks. Operative procedures performed were appendicectomy (95 per cent, 266 patients), right hemicolectomy with anastomosis (4 per cent, 10 patients), washout and drain (1 per cent, 4 patients) and other (0.4 per cent, 1 patient) with missing data for 5 patients (2 per cent) (*Table 1*). Operative duration was less than 90 minutes in 79 per cent (225 of 285 patients) of cases where antibiotics were unsuccessful compared with 77 per cent in the initial surgery group (1545 of 1997 patients) (*P* = 0.601).

More than a quarter of patients that had unsuccessful non-operative management did not have a CT (28 per cent, 80 of 284 patients). Of those that did, 91 per cent (186 of 204 patients) had CT features of appendicitis, meaning only 65 per cent of patients had confirmed appendicitis. Faecolithiasis was associated with unsuccessful non-operative management, present in 25 per cent (51 of 204) of patients with unsuccessful non-operative management and 18 per cent (139 of 777) of patients with successful non-operative management (*P* = 0.087). The presence of a faecolith was associated with a higher rate of perforated appendicitis and gangrenous appendicitis at operation when compared with patients who did not have a faecolith: 47 and 21 per cent *versus* 20 and 7 per cent respectively.

Histological examination of the appendix in patients with unsuccessful non-operative management showed acute appendicitis in 84 per cent (232 of 277 patients), chronic or previous appendicitis in 3 per cent (7 of 277), malignancy in 1 per cent (4 of 277), neuroendocrine tumour in 1 per cent (4 of 277), normal in 6 per cent (18 patients) and ‘other’ in 3 per cent (9 patients). The LOS in patients who had unsuccessful non-operative management was a median of 4 (i.q.r. 3–6) days. The outcomes of these patients are shown in *Table 2*.

**Table 2:** Outcomes in unsuccessful non-operative management patients

Outcome	Frequency (*n* = 286)
**Length of hospital stay (days)** *	4 (3–6)
**Intra-abdominal collection** †	
Managed with antibiotics alone	12 of 284 (4)
Managed with interventional radiology drain	1 of 286 (0.4)
**Postoperative ileus** ‡	20 of 284 (7)
**Wound infection**§	
Managed with antibiotics alone	10 of 284 (4)
Requiring incision and drainage	4 of 284 (1)
**HAP**	
Managed with oral antibiotics	1 of 284 (0.4)
Managed with intravenous antibiotics	2 of 283 (0.7)
**MI**	0 of 284 (0)
**DVT/PE**	0 of 284 (0)
**COVID-19**	6 of 284 (2)
**Death (30 days)**	1 of 284 (0.4)
**Unplanned level 2 of 3 care**	6 of 284 (2)
**Unplanned reattendances within 90 days**	63 of 274 (23)
**Any complications (collection, COVID-19, wound infections, HAP, MI, DVT/PE and death)**	28 of 283 (10)

Values in parentheses are percentages unless indicated otherwise;

*values are median (i.q.r.).

† Collection refers to an infected fluid collection or intra-abdominal abscess requiring treatment.

‡ Ileus was defined as a partial or complete non-mechanical blockade of the small intestine.

§Wound infection includes both superficial and deep incisional surgical-site infection, defined according to Centres for Disease Control and Prevention criteria as superficial as ‘involving only skin and subcutaneous tissues’ and deep as ‘involving deep structures such as fascia or muscle’. HAP, hospital-acquired pneumonia; MI, myocardial infarction; DVT/PE, deep vein thrombosis/pulmonary embolism.

### Matched outcomes

A comparison of propensity score-matched patients (1222 matched patients in each group by co-variables listed in Supplementary material online, *Table S1*) is shown in *Table 3*. Median LOS was shorter in the non-operative management group (2.5 (i.q.r. 1–4) *versus* 3 (i.q.r. 2–4) days, *P* < 0.001). There was no difference between operative and non-operative groups for risk of serious complications, including myocardial infarctions, venous thromboembolism, unplanned critical care admission or death. Hospital-acquired pneumonia (odds ratio 0.16; 95 per cent c.i. 0.05 to 0.45), wound infection (odds ratio 0.22; 95 per cent c.i. 0.12 to 0.42) and intra-abdominal collections (odds ratio 0.48; 95 per cent c.i. 0.32 to 0.74) were significantly lower in the non-operative group as opposed to matched operative management patients, giving a lower overall risk of complications (odds ratio 0.36; 95 per cent c.i. 0.26 to 0.50). There was an increase in unplanned reattendances and readmissions in the non-operative group (odds ratio 1.39; 95 per cent c.i. 1.11 to 1.75).

**Table 3:** Outcomes for propensity score-matched participants

Event	Operative management (*n* = 1222)	Non-operative management (*n* = 1222)	**Odds ratio or incident rate ratio** ^†^
**Unsuccessful non-operative management**	N/A	249 of 1222 (20)	
**Length of hospital stay (days)** *	3 (2–4)	2.5 (1–4)	**0.89 (0.83, 0.95)**
**Intra-abdominal collection**	68 of 1212 (6)	34 of 1216 (3)	**0.48 (0.32, 0.74)**
Managed with antibiotics	68 of 1212 (6)	31 of 1217 (3)	**0.43 (0.28, 0.67)**
Managed with interventional radiology drain	6 of 1222 (0.5)	5 of 1221 (0.4)	0.83 (0.25, 2.73)
**Postoperative ileus**	72 of 1212 (6)	22 of 1217 (2)	**0.28 (0.17, 0.46)**
**Wound infection**	52 of 1212 (4)	12 of 1216 (1)	**0.22 (0.12, 0.42)**
Managed with antibiotics	46 of 1212 (4)	11 of 1216 (1)	**0.24 (0.12, 0.46)**
Requiring incision and drainage	15 of 1212 (1)	5 of 1216 (0.4)	**0.33 (0.12, 0.92)**
**HAP**	25 of 1212 (2)	4 of 1217 (0.3)	**0.16 (0.05, 0.45)**
Managed with oral antibiotics	5 of 1212 (0.4)	1 of 1217 (0.1)	0.20 (0.02, 1.71)
Managed with intravenous antibiotics	22 of 1212 (2)	3 of 1217 (0.3)	**0.14 (0.04, 0.46)**
**MI**	1 of 1212 (0.1)	2 of 1216 (0.2)	2.00 (0.18, 22.1)
**DVT/PE**	5 of 1212 (0.4)	1 of 1217 (0.1)	0.20 (0.02, 1.71)
**COVID-19**	9 of 1212 (0.7)	10 of 1217 (0.8)	1.11 (0.45, 2.73)
**Death (30 days)**	4 of 1212 (0.3)	2 of 1217 (0.2)	0.50 (0.09, 2.73)
**Unplanned level 2/3 care**	13 of 1212 (1)	8 of 1217 (0.7)	0.62 (0.26, 1.48)
**Unplanned re-attendances within 90 days**	163 of 1171 (14)	216 of 1170 (18)	**1.39 (1.11, 1.75)**
**Any complications (collection, COVID-19, wound infections, HAP, MI, DVT/PE and death)**	141 of 1212 (12)	55 of 1215 (5)	**0.36 (0.26, 0.50)**

Values in parentheses are percentages unless indicated otherwise; bold text denotes values of statistical significance.

*values are median (i.q.r.).

† Values in parentheses are 95 per cent confidence intervals. HAP, hospital-acquired pneumonia; MI, myocardial infarction; DVT/PE, deep vein thrombosis/pulmonary embolism.

### Negative appendicectomy

Overall, the negative appendicectomy rate was 3 per cent (68 of 2274 patients): the appendix was histologically normal in 3 per cent (50 of 1994 patients) of those having initial operative management and 6 per cent (18 of 280 patients) of those who had unsuccessful non-operative management (*P* = 0.001). The majority of normal appendicectomies were performed in women: 72 per cent (36 of 50) in the operative group and 78 per cent (14 of 18) in the unsuccessful non-operative group.

### Cost analysis at 90 days

At 90 days, the operative management group incurred higher mean costs per patient than the non-operative group. The incremental cost per patient in the non-operative management group was −€1034 (95 per cent c.i. –€1201 to −€865), that is a saving of that amount. The results were robust to alternative assumptions made in the sensitivity analyses, including the potential for unmeasured confounding or the approach taken in the unit cost calculation (Supplementary material online, *Tables S2–S5* and *Fig. S3*). Costs were generally similar between the two groups across the resource-use categories. The main driver of differences in costs was the cost of appendicectomy in the primary admission, which was not offset by the costs accrued by patients in the non-operative management group who had appendicectomy in subsequent readmissions.

## Discussion

This large, multicentre cohort study shows that treatment of appendicitis with antibiotics can be successful in 80 per cent of patients by 90 days after presentation. Supported by high levels of CT scanning, non-operative management has fewer complications, results in less time in hospital and even if an interval appendicectomy is performed in some patients, the total costs per patient are lower compared with those for operative management.

The non-operative success rate of 80 per cent confirms results from similar trials that found antibiotics can avoid emergency surgery for the majority of patients with uncomplicated appendicitis (71 per cent and 84 per cent)^19^^,^^20^. The non-operative group was unselected, included nearly double the number of patients of the CODA trial, five times that of the APPAC trial and represents the largest ever series of adult appendicitis patients managed non-operatively^19^^,^^20^.

There were fewer complications and a shorter LOS in the non-operative group, a finding paralleled in other studies and maintained in those which report long-term follow-up^21^^,^^22^. There was little evidence that failure of non-operative management led to more complicated operations or worse outcomes; rates of laparoscopically completed appendicectomy and operative durations were not significantly different from those for patients who were treated operatively from admission. Of the one in five who failed non-operative management, the LOS was 1 day longer than that for those who had initial operative management.

Even accounting for those who required interval appendicectomy, the average cost per patient was lower in the non-operative group with a mean reduction of –€1034 (95 per cent c.i. –€1201 to −€865). These findings are consistent with other cost-analysis studies, although previous cost-analysis comparison was against open appendicectomy^23^^,^^24^. In practice, the cost of laparoscopic appendicectomy varies between surgeons^25^, dependent primarily on the use of energy or stapling devices for appendix base closure. To account for this the unit cost of laparoscopic surgery was varied over values compatible with alternative appendiceal stump closure techniques in sensitivity analysis. Sensitivity analysis also accounted for laparoscopic appendicectomy being performed at usual rates (95 per cent)^10^ rather than the reduced rate seen during the pandemic.

Current guidelines recommend that a faecolith on imaging should be treated with surgery^9^. As a result, clinical trials have excluded patients with a faecolith^2^^,^^19^. Despite this 27 per cent of patients with an identified faecolith were managed non-operatively. However, a higher percentage of patients with unsuccessful non-operative management had a faecolith present and those with a faecolith who had unsuccessful non-operative management had more severe appendicitis on subsequent operation, with higher rates of gangrene and perforation than those without^19^. This will be explored further in the planned 1-year follow-up.

Potential missed appendiceal malignancy is a clinical concern of non-operative management. This study found four cases of malignancy and three neuroendocrine tumours in the initial non-operative management group, and an overall rate of malignancy of 1.3 per cent, consistent with the population reported incidence of 0.5–1.7 per cent^26^. Known risk factors for appendiceal malignancy are age, appendiceal dilatation on CT and complicated appendicitis^26^^,^^27^. Again, longer follow-up will give a clearer picture of this risk.

In addition to providing assessment of potential markers of malignancy, CT scanning is sensitive for diagnosing appendicitis^28^ and the higher use in this study (70 per cent *versus* 18 per cent of patients presenting with right iliac fossa pain in 2019)^10^, is likely to have directly influenced the low overall negative appendicectomy rate (3 *versus* 20 per cent previously reported by the RIFT Study Group)^10^. However, CT involves ionizing radiation and consideration should be given towards contrast-enhanced low-dose CT scanning in younger adults^29^^,^^30^.

Following the landmark Montgomery UK Supreme Court ruling in 2015, the concept of informed consent has been re-evaluated^31^. Surgeons are required to present all treatment options including the benefits, risks of harm and likelihood of success for each option^32^. As part of these legal requirements, non-operative as well as operative management should form part of every surgeon’s discussion of treatment options for acute appendicitis. Previously clinicians had cited concerns over the external validity of RCT evidence as a reason not to offer non-operative management. This study demonstrates the first time non-operative management has been implemented by UK surgeons and used on a wide scale in the surgical population.

As an observational study, there was no standardized antibiotic protocol and the decision to proceed with surgery was at the clinician’s discretion. There is inherent bias when implementing any novel management, and it is challenging to capture changes that result from a different consultant taking over the patient’s care, therefore there is the possibility of residual confounding. A large proportion of patients in the antibiotic group were diagnosed clinically, meaning that a presumed diagnosis of appendicitis was given to some patients. This could be a confounder for how many patients had truly unsuccessful non-operative management, particularly those with normal appendix histology. CT was not used universally as in previous RCTs. This is a strength as resource constraints in the National Health Service would probably prevent universal CT scanning if non-operative management were adopted in the future. However, this may have contributed to the 6 per cent negative appendicectomy rate in the group managed non-operatively.

## The COVID: HAREM collaborators group

N. Kulkarni; I. Pereira; S. Barlow; S. Vanniasegaram (Lincoln County Hospital, Lincoln); F. Loro; N. S. Blencowe; B. E. Zucker; A. Tyler (Bristol Royal Infirmary, Bristol); M. Hollyman; A. Kosti; M. Wijeyaratne; T. Badenoch (Musgrove Hospital, Taunton); S. Wheatstone; M. Jaffer; H. Gerretsen; R. Menon (Guys and St. Thomas Hospital, London); M. S. Sajid; L. Kennedy; A. Malik; A. Nada; K. Ray; M. Khan (Royal Sussex County Hospital, Brighton); M. Varcada; F. Froghi; A. Khalil; D. Kyprianou (Royal Free Hospital, London); N. Tewari; D. R. Sarma; M. Baig; S. Sood; E. Yu Wen Ng; V. Ng; T. Shortland; G. Marangoni; S. Khan; J. Ahmad (University Hospital Coventry, Coventry); S. Brown; C. Steele; A. Pannu (Sheffield Teaching Hospital, Sheffield); E. Gemmill; H. Boyd-Carson; P. Herrod; S. Singh Shari; M. J. S. Mohammed; V. Narbad; N. Hanbali; A. Kushairi (Kings Mill Hospital, Mansfield); M. A. Mathew; C. Downey; A. Alamassi (Airedale NHS Foundation Trust, Steeton); T. Wheatley; K. Emslie; B. Alcocer; S. Lau (Derriford Hospital, Plymouth); R. Morgan; T. Gala; S. Ibrahim; M. Stephanos; R. Mithany; M. Abdelkarim; G. Venkatesan; A. Aqsalan (Glan Clwyd Hospital, Rhyl); J. Taylor; M. Fok; A. Kattakayam; K. Rajput (Liverpool University Hospitals NHS Trust, Liverpool); K. Bevan; H.-K. Kim; L. Salih; R. Sabaratnam (Bedford Hospital, Bedford); M. Creanga; A. Shafi; J. Law; M. Elniel; M. Walley; S. Ayyar (Pennine Acute Hospitals NHS Trust, Oldham),; J. Cornish; N. Reeves; N. Mowbray; I. Mayo; M. Shinkwin (University Hospital of Wales, Cardiff); E. Chohda; W. McCaughran; E. Beck; S. Garikipati (Whittington Hospital, Whittington); B. E. Lovett; F. Alkistawi; S. Franklin; C. Hadjitoffi; A. Uddin (Basildon University Hospital, Basildon); P. K. Patel; S. Handa; J. Parker; D. Littlehales (Furness General Hospital, Furness); A. P. Belgaumkar; B. Oyewole; P. Narayan; Z. Elahi; A. Gaukroger (Surrey and Sussex Healthcare NHS Trust, Surrey); D. F. J. Dunne; G. E. Nita; R. D. Baron; D. Sochorova; P. Szatmary; S. A. K. Gahunia; A. J. Thomas; K. S. Mann (Royal Liverpool University Hospital, Liverpool); M. McFall; N. Farkas; H. Siddig (Worthing Hospital, Worthing); J. Camilleri-Brennan; D. Rutherford; M. Wilson; E. Massie; K. McGivern; J. McGuckin; C. McKee (Forth Valley Royal Hospital, Larbert); S. Marinos-Kouris; E. Gammeri; N. Patel; G. Cillo; A. J. Baldwin; T. Magro (Stoke Mandeville Hospital, Aylesbury); K. Krishna; J. Olivier; N. Anyaugo; K. Philip (Weston General Hospital, Weston-super-Mare); L. Pearce; A. Al-Amin; M. Thomas; I. Anderson; R. Clark (Salford Royal Hospital, Salford); M. Basamh; S. M. Navaratnam; A. Saunt; B. Bekhyat Karki; H. Jeong; B. Singh; A. Rajendirin; K. Boyle; S. Fahmy; J. H. Couch; H. Z. Butt (University Hospitals of Leicester NHS Trust, Leicester); G. Tierney; J. N. Lund; H. Javanmard-Emamghissi; B. Doleman; C. Hope; A. Gowda; D. Photiou; F. Malcolm; P. Daliya; N. Rye; Z. Chia; F. Anis (Royal Derby Hospital, Derby); P. Thomas; T. Urbonas; D. Centea; N. Husain (Burton Hospital, Burton); S. Moug; A. Ingham; N. Galbraith (Royal Alexandra Hospital, Glasgow); R. Alexander; C. Bisset (Inverclyde Hospital, Greenock); R. Clifford; L. Dickerson (The Countess of Chester Hospital, Chester); S. Lockwood; J. Johnston (Bradford Royal Infirmary, Bradford); R. Guy; T. Majeed; R. Young; S. Shamim; M. Mesri (Arrowe Park Hospital, Birkenhead); R. Date; M. P. Chaudhury; G. Zambas (Lancashire Teaching Hospital, Preston); R. Patel; S. Lewis; A. T. Eigbadon; D. Thakrar; E. Karamitsou; Y. Oyeyipo; U. Nadeem; S. Ndlovu; A. Fanshawe (Northwick Park Hospital, London); N. Henderson; C. Payne; D. Porter (Ninewells Hospital, Dundee); A. Brooks; R. X. N. Lee; J. Jackman; A. J. Morton; O. Ebunoluwa Oyende; D. Worku; A. Koh; T. Kanani; J. Blackwell; M. Shaw; C. Lloyd-Lewis; L. Blackburn; A. Adiamah (Queens Medical Centre, Nottingham); S. Shaikh; M. Ghazanfar; M. Elhusseini; A. Abdelhamid; J. Eley; A. Nassar (aberdeen Royal Infirmary, Aberdeen); R. Nunn; A. Gales; E. Farinella; Z. Mahmood; T. Policastro (Lister Hospital, Stevenage); N. M. Bagnall; U. Blyth (Victoria Royal Infirmary, Newcastle); R. J. McGregor; D. Damaskos; M. Drogouti; Z. Tuharska (Royal Infirmary of Edinburgh, Edinbugh); J. Davies; J. M. Bennett; R. Antakia; J. R. O’Neill; R. H. Hardwick; N. Fearnhead; A. Xanthis; F. Georgiades; V. Hudson; J. Ashcroft; A. A. Singh (Addenbrookes Hospital, Cambridge); S. M. U. Kabir; H. Huan; M. Sugrue (Letterkenny University Hospital, Letterkenny); M. Riera; J. Chang; A. Omosebi; E. Rigby; L. Kim; S. Ali; Z. Gates; H. Alasa; J. Y. N. Bo; A. Gangwar (Shrewsbury Hospital, Shrewsbury); L. Osborne; B. Perakath (Dr Gray's Hospital, Elgin); M. Chandarana; M. Galea; A. Luhmann (Victoria Hospital, Kirkcaldy); O. Ryska (Royal Lancaster Infirmary, Lancaster); F. Searight; C. McCoss; B. Weber (Gilbert Bain Hospital, Lerwick); M. Sallam; R. Patel; M. Bignell; G. Bond-Smith; C. Lewis (John Radcliffe Hospital, Oxford); G. Williams; H. Whewell; L. Smith; R. Ooi; A. Powell-Chandler; A. M. Tang (Royal Gwent Hospital, Newport); S. K. Richards; D. B. Thompson; R. Cross (Royal United Hospital, Bath); J. van Dellen; V. Alberto; S. Shirazi; H. Arang; N. Rahman (Croydon University Hospital, Croydon); E. Monaghan; K. Dodds; O. Babalola; P. Airhunmwunde; C. Chinaka (South West Acute Hospital, Enniskillen); I. Wijetunga; T. Kidd; K. Nambiar; C. E. Ng; T. Collier; B. Ibrahim; K. Khan (University Hospital North Durham, Durham); K. Sriskandarajah; T. Pelly; J. Vance-Daniel (Kingston Hospital, Kingston); P. Nastro; A. Khan; O. Ekowo; A. Devadoss (Darent Valley Hospital, Dartford); P. D. Rao; K. Bateman; A. Gavrila (Glangwilli General Hospital, Glangwilli); E. Hannan; D. Winter; S. Martin; R. Kennelly; A. Hanly (St Vincent's University Hospital, Dublin); M. I. Aslam; V. Amin; R. Wilkins; S. Zafar; C. Konstantinou; S. Mcdonald; A. Baker; A. Fardie (Warwick Hospital, Warwick); A. Hill; J. De Marchi; S. O'Grady (Beaumont Hospital, Dublin); G. Faulkner; H. Sekhar; M. Martinez-Iglesias; C. Alexander; E. Lawrence; S. Argyropoulos (Royal Bolton Hospital, Bolton); G. Williams; S. Bhasin (Royal Wolverhampton Hospital, Wolverhampton); M. Paduraru; K. Pawelec; S. Bylapudi (Milton Keynes University Hospital, Milton Keynes); H. Byrne; E. R. Da Silva Bento; F. Zahari; F. Roslan; M. Rao (Pilgrim Hospital, Boston); S. Hudson-Phillips; C. Kenington; S. Tellman; P. Abraham; A. Dhillon; Z. Vinnicombe (St George's Hospital, London); M. Giles; M. Abbakar; N. Khadem; E. Buckley; L. Macdonald; J. Norman; R. Bond (York NHS Foundation Trust, York); T. White; T. Gana; S. Kotecha; S. Rajain (Chesterfield Royal Hospital, Chesterfield); S. Ahmad; B. Wadham; L. Hancock (Wythenshaw Hospital, Manchester); A. Liyanage; I. Dorrington; A. Mian; R. Y. Satchidanand; C. Weerasinghe (Southport Hospital, Southport); K. J. Etherson; H. Hidayat; M. Bhandari; A. Agarwal (The University Hospital of North Tees, Stockton-on-Tees); J. Sagar; S. Kudchadkar; A. Ghosh; N. Cirocchi; A. Rai; O. AlHabsha; S. S. Mujtaba (Luton and Dunstable Hospital, Luton); F. Ejtehadi; I. Warrag; B. Ivanov; J. Refson; C. Boateng (The Princess Alexandra Hospital, Harlow); R. Madani; M. M. Buhsk; D. Kesharwani; L. Kumar; V. Prakash; S. Zulfiqar; A. Jayakumar(St Peter's Hospital, Chertsey); A. Payne; C. Davies (Barnsley Hospital, Barnsley); R. Buhain; D. Osilli; T. Rashid; I. Elzayat (Queens Hospital, Romford); V. Kanakala; E. J. Nevins; A. Madhavan; E. Oates; K. France; S. Cowie (James Cook University Hospital, Middlesbrough); J. Bowen; Y.-J. Nam; M. Bradbury; V. Mitchell (Torbay Hospital, Torbay); S. M. Mirza; M. M. Raiz; E. Weatherstone; R. Wilson (Hinchingbrooke Hospital, Hinchingbrooke); K. Sasapu; M. M. A. Rahman; E. Chan; K. Y. Ko; M. Sharman (Diana Princess of Wales Hospital, Grimsby); K. Thiruppathy; J. Hodgkinson; R. Chadha; T. Pilpel; J. Dale (Royal Berkshire Hospital, Reading); N. Carter; A. Botros; I. Bondoqa; S. Sandabah; K. Sherwood (Queens Alexandra Hospital, Portsmouth); R. Harries; L. Hurt; R. Egan; L. Gauntlett; V. Bevan (Swansea Bay Hospital, Swansea); M. Vipond; P. Ireland; S. Granger; R. Preece (Gloucestershire Hospitals NHS Foundation Trust, Gloucester); D. Frith; J. Eves; A. Abuown (Imperial College Healthcare NHS Trust, London); J. Apollos; A. Macleod; N. Hemadasa (Dumfries & Galloway Royal Infirmary, Dumfries); C. McNaught; R. Mir; G. Cuthbert (Harrogate District Hospital, Harrogate); C. Valero (Norfolk and Norwich University Hospital, Norwich); D. Williams; M. Fakhrul-Aldeen; K. Willis; L. Kelly (North Devon Distric Hospital, Barnstaple); D. Lawes; L. Poynter; H. Knowles; S. Saeed; M. Shehata; I. Rafiq; M. Boshnaq; F. Ayoub (Tunbridge Wells Hospital, Royal Tunbridge Wells); A. Mcnair; D. J. Pournaras; S. Lawday; R. Martin; H. Cohen; M. Okocha (Soutmead Hospital, Southmead); K. Shalli; M. Chin; S. Joliffe (Wishaw General Hospital, Wishaw); F. Taylor; E. O. Argyriou; M. Dornseifer; E. Schembari; S. Surandran; L. Roberts; G. Kakaniaris (Whipps Cross University Hospital, London); E. Mallidis; G. Karagiannidis (Ipswich Hospital, Ipswich); F. Youssef; A. Chan; C. Macutkiewicz; M. Davenport; S. Hodge; A. Clarke (Manchester Royal Infirmary, Manchester); G. Branagan; R. Thakkar (Salisbury District Hospital, Salisbury); C. Harris (Northumbria Specialist Emergency Care Hospital, Cramlington); C. Brown; M.-C. McGuigan (Queen Elizabeth University Hospital, Glasgow); I. Alam; K. Wang; F. Artemis (Royal Albert Edward Infirmary, Manchester).

## Supplementary Material

znab287_Supplementary_Data

## Notes

Members of The COVID: HAREM (Had Appendicitis and Resolved/Recurred Emergency Morbidity/Mortality) Collaborative Group are co-authors of this study and are listed under the heading Collaborators.

Presented at the Association of Surgeons of Great Britain and Ireland Conference, May 2021
