# Supplementary material for: Antibiotics as first-line alternative to appendicectomy in adult appendicitis: 90-day follow-up from a prospective, multicentre cohort study
Source: Br J Surg. 2021 Sep 3;108(11):1351–9. doi: 10.1093/bjs/znab287 (PMC8499866; doi:10.1093/bjs/znab287)
Supplement: znab287_Supplementary_Data [file znab287_supplementary_data.docx]

**Supplementary information**

**Cost analysis up to 90 days**

***Overview***

The cost analysis undertook a time horizon of 90 days, and was undertaken from the hospital perspective. The costs included were those pertaining to the index hospital admission, and any subsequent readmissions. Resource use categories were defined a priori and include those where differences between groups are likely to drive incremental costs. These are the duration of hospital admission including days in critical care and ward; duration and choice of surgical approach (open or laparoscopic); the use of antibiotics; the use of imaging; and any subsequent readmissions. Total costs at 90 days were calculated by combining resource use data at the patient level with unit costs at 2018/19 prices (£ GBP). The results were subjected to extensive sensitivity analyses, including the potential for unmeasured confounding and the approach taken in the unit costs analysis. The specific resource use categories were:

1. *Surgical procedures: open and laparoscopic appendicectomy*

The cost of delivering surgery included the costs of the consumables, staffing, overheads and use of level 2 (HDU) and 3 (ICU) post-operative care for both laparoscopic and open surgery. Patient level data on the duration of the procedure, route of operation (open or laparoscopy), grade level of the staff and use of HDU and ICU post-operative care were retrieved from the COVID:HAREM database. The costing of consumables was informed by HAREM hospitals’ pharmacy departments and based on standard use of equipment for appendicectomy (i.e. laparoscopic instrument pack, ports, graspers and scissors, ligating loop, dressings and sutures, for laparoscopic appendicectomies; and universal instrument pack, sutures, diathermy and dressings, for open appendicectomies). Use of general anaesthetics was assumed for patients receiving both laparoscopic and open procedures. Staffing levels in the main analysis were informed by expert opinion and included: two surgeons (consultant and registrar), two anaesthetists (consultant and registrar) and five nurses (scrub and circulating). Data on level of post-operative care and use of post-operative HDU and ICU care was retrieved from COVID:HAREM database. For stays in HDU and ICU, a duration of stay of 2 and 3 days, respectively, was assumed and costed accordingly. Sensitivity analyses were conducted to assess the robustness of the results to higher/lower costs associated with the intervention, including staffing levels, costs of consumables and costs of overheads.

1. *Antibiotic therapy*

The cost of antibiotic therapy associated with delivering the study interventions for both comparators, operative and conservative management, was derived. For each patient, resource use data on duration of antibiotic therapy and route (intravenously “IV” or oral “PO”) was retrieved from the COVID:HAREM database. Where multiple antibiotics were prescribed, information of duration and route of administration for each medication was collected and costed separately. Expert clinical opinion was sought to inform standard use and dosage of pre-, intra- and post-operative IV or PO antibiotic therapies that are routinely prescribed for treating acute appendicitis in NHS hospitals.

1. *Diagnostic imaging*

The costing of diagnostic imaging strategies in both treatment groups considered use of Computed Tomography (CT), Magnetic Resonance Imaging (MRI) and Ultrasound (USS) scan. Resource use data of each of form of imaging was retrieved from the COVID:HAREM database. Informed by expert opinion, a total duration of the procedure of less than 20 minutes was assumed for USS. For patients with MRI and CT scans, no use contrast was assumed. Where multiple forms of imaging were recorded, each procedure was costed separately.

1. *Length of stay*

The use of hospital resources in the primary admission was derived from the COVID:HAREM database. For the index admission, the length of stay in general medical wards was calculated as the total duration in days from admission until discharge or death, subtracting the time the patient spent in post-operative HDU and ICU care.

1. *Re-admissions*

A hospital readmission was defined as a further hospital admission within 90 days following ultimate hospital discharge from the primary admission. All readmissions occurring within 90 days after discharge were costed. For patients who did not receive surgery, the cost of readmission was derived using information on whether it was an overnight stay (including elective and non-elective inpatient admissions and taking into account the duration of the readmission), or a day attendance (e.g. follow-ups in surgical assessment units). Readmission costs for these patients include, for example, those associated with assessments of resolution of the condition using diagnostic imaging or blood tests.

For patients who received surgery in the readmission, the aggregated readmission cost was derived taking into account the type of surgery, the reason for readmission and the existence of preceding surgeries of the appendix. Where information on the type of procedure was available, we derived the relevant cost for the readmission from the NHS 2018/2019 National Schedule of Reference Costs^1^. We took great care to accurately derive the readmission cost where information on the type procedure was not available. We defined two broad readmission cost categories depending on whether the operation involved major/complex (e.g. reopening and exploration of the abdomen) or minor/intermediate (e.g. irrigation of peritoneal cavity) abdominal surgery. The readmission costs for patients in both categories was derived from the NHS 2018/2019 National Schedule of Reference Costs^1^. For readmissions preceded by appendicectomies or right hemicolectomies, we assumed the cost to be that of readmissions involving minor therapeutic or diagnostic procedures. For the remaining appendix-related admissions that were not preceded by surgeries of the appendix, we made the assumption that 50% of these involved major or complex general abdominal procedures, whereas the rest were assumed to be minor or intermediate procedures. The robustness of the results to these assumptions was assessed through sensitivity analyses.

***Unit costs***

The unit costs required for valuing the resource use data were taken from national unit cost databases and are listed in Table S1 below. Direct costs associated with USS, MRI and CT scans were retrieved from the 2018/2019 National Schedule of Reference Costs^1^. Other costs associated with other forms of diagnosis (e.g. blood tests) were not considered in the analysis. Open and laparoscopic appendicectomies were costed assuming same staffing levels, overheads and bed-day cost of HDU and ICU wards, but different use of consumables. Overheads costs were retrieved from ISD Scotland’s 2018/2019 Theatre Costs^2^. The cost of the consumables used in open and laparoscopic appendicectomies was informed by the pharmacy department at one of the HAREM study’s hospitals. The unit costs associated with staff were retrieved from a recommended published source for Health and Social Care costs^3^. Unit costs for the six most common antibiotic drugs in the study – i.e. Co-amoxiclav; Amoxicillin, Metronidazole, Cefuroxime, Gentamicin and Tazocin–, were retrieved from the British National Formulary (BNF) using indicative quantities/doses informed by clinical judgement^4^. The use of these drugs was costed separately and considering the exact duration of the treatment and the route of administration (intravenously or oral). Costs of general anaesthetics were also retrieved from the BNF. Cost of on bed-day in the general medical ward, SAU and HDU and ICU care was retrieved from the 2018/2019 National Schedule of Reference Costs^1^. Likewise, readmission costs were derived extracted from the 2018/2019 National Schedule of Reference Costs^1^.

***Assumptions considered in the base case analysis and corresponding sensitivity analyses***

Table S4 lists the main assumptions made in the base case scenario, and how each of them was relaxed in sensitivity analyses. The results of the sensitivity analysis are reported in Figure S1 as mean incremental costs with corresponding 95% CIs.

**1. Overhead costs for appendicectomies in primary admission.** In the base case, overhead costs for appendectomies were taken from ISD Scotland 2018/2019 Theatre Costs, for the specialty of gastroenterology. These can account for up to 34% of the total operative cost, but this proportion varies significantly across trusts. In the sensitivity analysis we varied the cost over a range of possible higher and lower values compatible with the data from other NHS trusts in ISD.

**2. Consumable costs for laparoscopic appendicectomies in primary admission.** In the base case, unit cost of the consumables for laparoscopic appendectomies was informed by one of HAREM hospitals’ pharmacy department. In practice, the cost for the laparoscopic set can vary across sites, being the chosen technique for appendiceal stump closure a key driver of the operative costs. In the sensitivity analysis we varied the unit cost of laparoscopic surgery over values compatible with alternative appendiceal stump closure techniques.

**3. Proportion of laparoscopic appendicectomies in primary admission.** In COVID:HAREM, about 70% of the surgeries performed in the primary admission were laparoscopic appendicectomies. Recent studies have shown that the rates of laparoscopic appendicectomies are much higher in England^6,7^. In the sensitivity analysis we assumed 95% of the appendicectomies to be performed laparoscopically.

**4. Theatre staff time in primary admission**. In the base case, we assumed that two surgeons (consultant and registrar), two anaesthetists (consultant and registrar) and five nurses (scrub and circulating) are in the operating room throughout the duration of the intervention. Staffing levels might depend on the context, including whether the operation was performed during or after the COVID-19 restrictions were in place, whether it was a daytime or night operation or even across healthcare providers. In the sensitivity analysis we varied the number of nurses (over 3-7) in order to consider alternative values that may apply in different contexts.

**5. Assumption about readmission costs.** The lack of information on the operations performed in some readmissions required making assumptions about the expected costs associated to them. In the sensitivity analyses we varied the cost of these procedures over reasonable values compatible with different forms of surgery in appendix-related admissions.

**6. Distributional assumption for costs.** The base case assumed that costs were normally distributed when reporting the 95% CIs around incremental costs. In sensitivity analyses we assessed the robustness of the results of the analysis to alternative distributional assumptions. Because costs have a right-skewed distribution, following methodological guidance, the sensitivity analysis considered a Gamma distribution.

**7. Confounding adjustment.** In the base case, incremental costs were reported for the propensity-score matched study population. In the sensitivity analysis, we added a regression adjustment model to further minimise the risk of confounding in the analysis. Covariates used for adjustment are the patients’: age, sex, Rockwood clinical frailty index, BMI, use of diabetes medications, COPD, smoking history, MI history, use of immunosuppressive medications, active cancer and dementia.

**References:**

1 Department of Health, NHS England, and NHS Improvement . Reference Cost Collection: National Schedule of Reference Costs, 2018–19 [https://www.england.nhs.uk/national-cost-collection/]Accessed on 30 Nov 2020.

2 Information Services Division Scotland (ISD). Theatres Costs-Detailed Tables - R142X. [http://www.isdscotland.org/Health-Topics/Finance/Costs/Detailed-Tables/Theatres.asp] Accessed on 30 Nov 2020.

3 Curtis, L. Unit Costs of Health and Social Care 2019. [http://www.pssru.ac.uk/project-pages/unit-costs/2012/]. Accessed on 30 Nov 2020.

4 British National Formulary. BNF. 78London: British Medical Association and the Royal Pharmaceutical Society of Great Britain. [http://www.ppa.org.uk/ppa/edt_intro.htm]. Accessed on 30 Nov 2020.

5 Clement KD, Emslie K, Maniam P, Wilson MSJ. What is the Operative Cost of Managing Acute Appendicitis in the NHS: The Impact of Stump Technique and Perioperative Imaging. World J Surg. 2020 Mar;44(3):749-754. doi: 10.1007/s00268-019-05306-2. PMID: 31773223.

6 Ventham NT, Dungworth JC, Benzoni C. Transition towards laparoscopic appendicectomy at a UK center over a 7-year period. Surg Laparosc Endosc Percutan Tech. 2015 Feb;25(1):59-63. doi: 10.1097/SLE.0000000000000017. PMID: 24732741.

7 Currow C, Patel K, Askari A, Rabie M, Aly M, Aker M; Surgical Trainees East of England Research (STEER) Collaborative. Current technical surgical practice of emergency appendicectomy: a cross-sectional survey of surgical registrars in the UK. Ann R Coll Surg Engl. 2020 Oct;102(8):606-610. doi: 10.1308/rcsann.2020.0123. Epub 2020 Jun 5. PMID: 32501113; PMCID: PMC7538747.

**Supplementary Figure 1. Violin plots before propensity score matching**

***
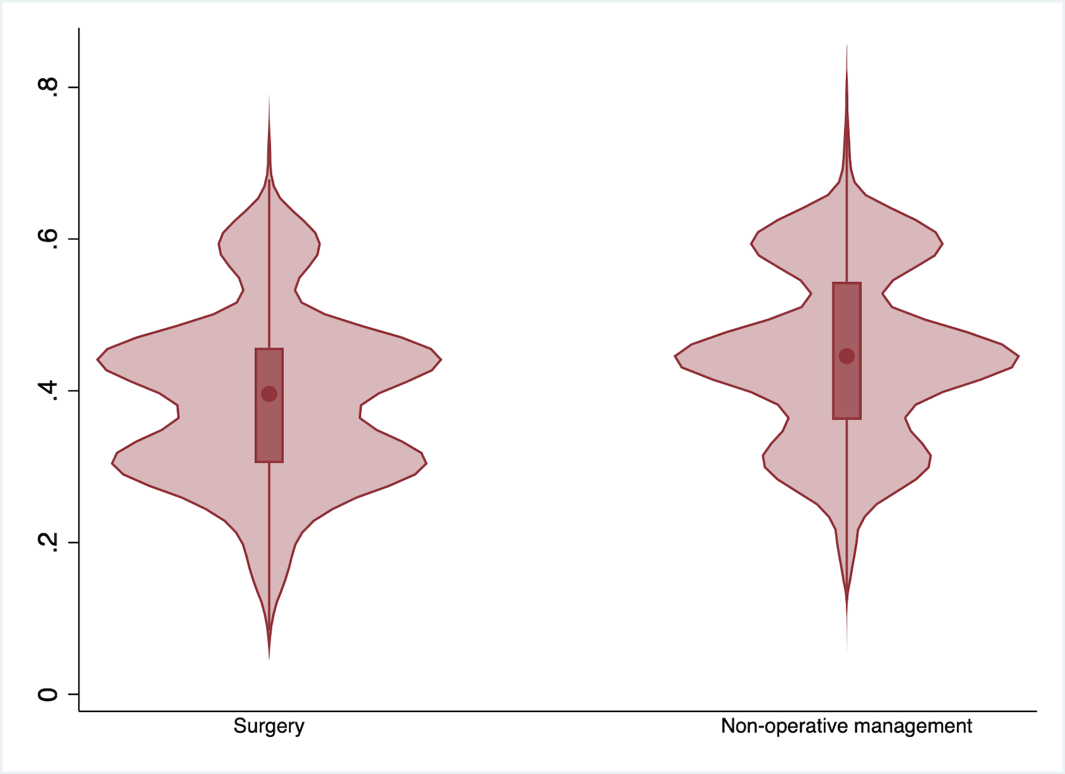
***

**Supplementary Figure 2. Violin plots after propensity score matching**

**
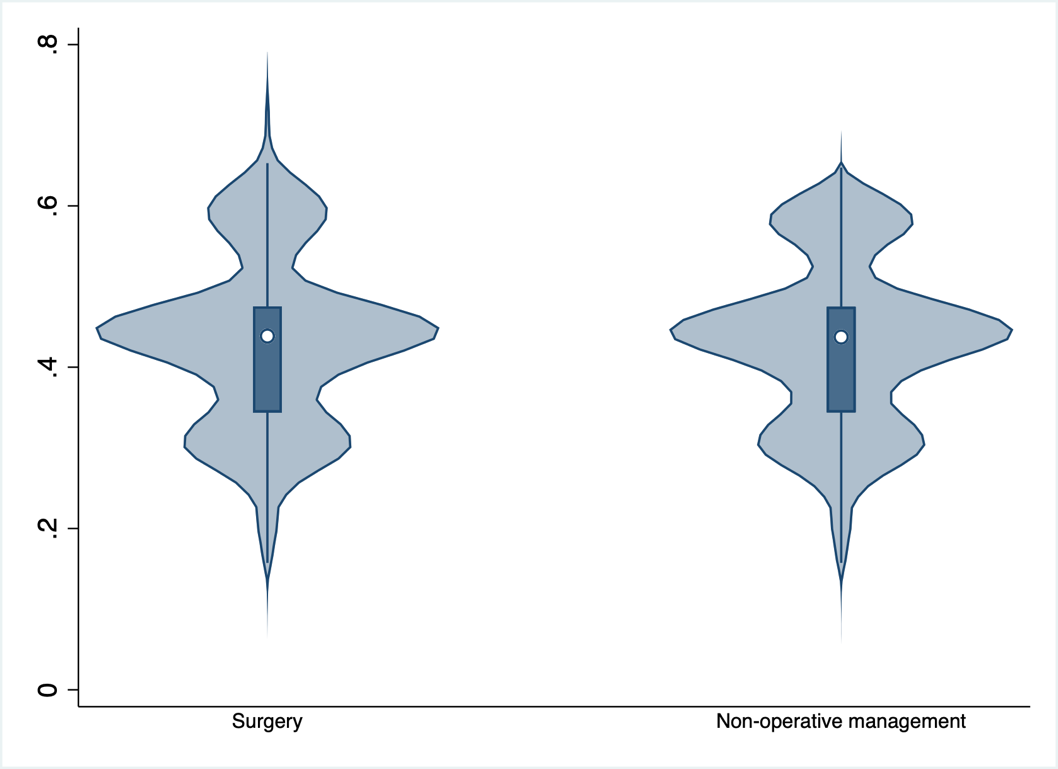
**

**Supplementary Figure 3. Sensitivity analysis: Incremental cost within the first 90 days (95% Confidence Interval), under the base case and alternative assumptions.**

**
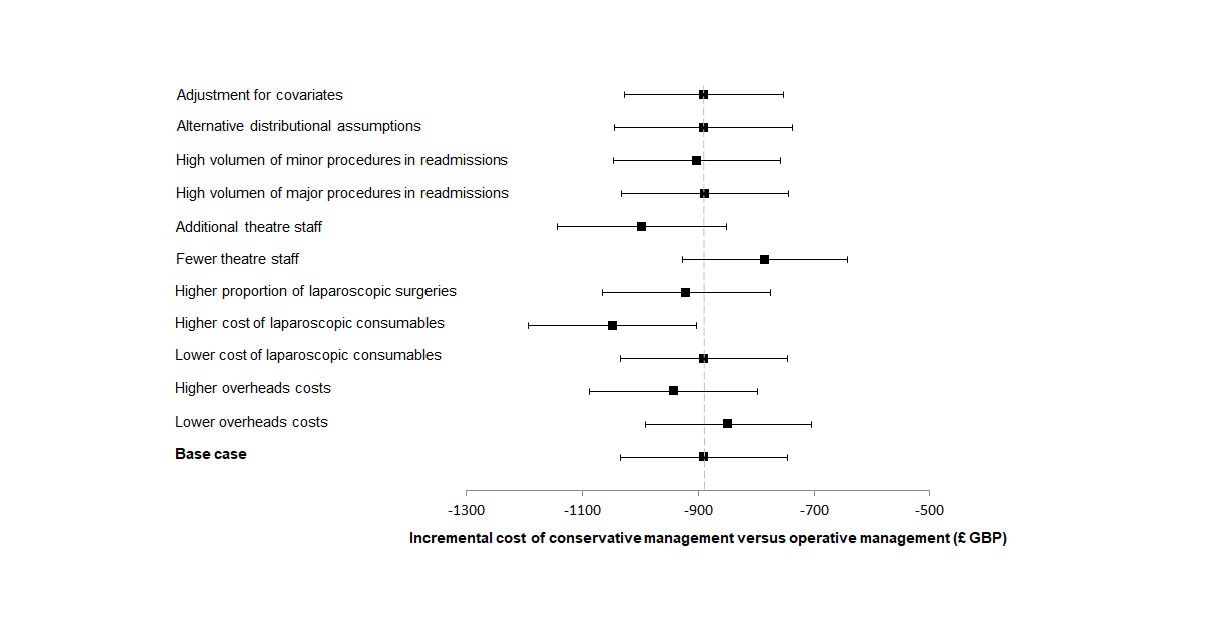
**

**Supplementary Table 1: Patient characteristics for propensity score matched participants.**

CRP was used as a marker of severity. Following this, there was no significant difference in participants who were febrile (SD=0.07; p=0.094) or tachycardic (SD=0.03; p=0.567).

| Event / Total (%) or Median [IQR] | Operative management (n=1222) | Non-operative management (n=1222) | Standardised difference (SD) (P value)^†^ |
| --- | --- | --- | --- |
| Age | 36 [27-51] | 35 [26-52] | 0.02 (0.308) |
| Female | 603/1222 (49) | 592/1222 (48) | 0.02 (0.686) |
| Body Mass Index  <20  20-25  25-30  30-35  35+ | 66/1222 (5)  487/1222 (40)  413/1222 (34)  179/1222 (15)  77/1222 (6) | 87/1222 (7)  454/1222 (37)  423/1222 (35)  166/1222 (14)  92/1222 (7) | 0.1 (0.201) |
| Co-morbidity  Chronic obstructive pulmonary disease  Myocardial infarction  Diabetes | 26/1222 (2)  34/1222 (3)  47/1222 (4) | 28/1222 (2)  33/1222 (3)  48/1222 (4) | 0.01 (0.891)  0.005 (1.000)  0.004 (1.000) |
| Adult Appendicitis Score group  Low Risk  Intermediate Risk  High Risk | 246/1222 (20)  691/1222 (57)  285/1222 (23) | 236/1222 (19)  703/1222 (58)  283/1222 (23) | 0.02 (0.858) |
| C-reactive protein on admission | 36 [9-92] | 38 [11-95] | 0.03 (0.295) |

**†** P values of significance are highlighted in bold

**Supplementary Table 2: Resource use and costs (€) to 90 days by group**

| **Cost component** | **Conservative management group (N=1222)** | **Operative**  **management group (N=1222)** |
| --- | --- | --- |
| **Primary admission** | | |
| Diagnostic imaging, n (%)  Cost per patient (€): mean (SD) | 1,064 (87)  78 (36) | 1,015 (83)  74 (38) |
| Days of antibiotic therapy: mean (SD)  Cost per patient (€): mean (SD) | 6.20 (4)  26 (35) | 3.63 (4)  23 (43) |
| Inpatient days in general ward (days): mean (SD)  Cost per patient (€): mean (SD) | 3.08 (3)  1,341 (1414) | 3.46 (4)  1,507 (1527) |
| Operations in primary admission^†^: n (%)  Cost per patient (€): mean (SD) | 182 (15)  224 (711) | 1,199 (98)  1,260 (748) |
| **Re-admissions** | | |
| Number of readmissions, n (%)  Cost per patient (€): mean (SD) | 447 (37)  386 (896) | 246 (20)  225 (995) |
| **Total cost per patient (€): mean (SD)** | *2,055 (2001)* | *3,089 (2216)* |
| **Incremental cost (95 per cent c.i.)** | *-1034 (-1201; -865)* | |

*Mean calculations were done using total number of patients in the group. **^†^**98% of the operations performed were appendicectomies; open and laparoscopic appendicectomies were costed separately.

**Supplementary Table 3: Unit costs (€)**

| **Item** | **Unit cost (€)**  **2018/19** | **Source** | | **Assumptions** |
| --- | --- | --- | --- | --- |
| **Diagnosis in primary admission (per patient)** | | | | |
| Diagnostic USS | 60 | NHS Reference Costs^1^ | | Ultrasound Scan with duration of less than 20 minutes, without Contrast |
| Diagnostic CT | 90 | NHS Reference Costs^1^ | | Computerised Tomography Scan of One Area, without Contrast, 19 years and over |
| Diagnostic MRI | 140 | NHS Reference Costs^1^ | | Magnetic Resonance Imaging Scan of One Area, without Contrast, 19 years and over |
| **Surgery in primary admission** | | | | |
| Overheads (per hour) | 235 | ISD Scotland^2^ | ISD Scotland Theatre 2018/2019 costs – sheet “R142X: “Theatre direct costs per hour, by specialty” and sheet “R141X: Theatre – Running costs”: Direct allocated costs (overheads) (total costs-direct staff costs-direct supplies costs) for gastroenterology. | |
| Consultant surgeon (per hour) | 126 | PSSRU^3^ | Hospital-based doctors – consultant surgeon | |
| Registrar surgeon (per hour) | 55 | PSSRU^3^ | Hospital-based doctors – registrar | |
| Consultant anaesthetist (per hour) | 126 | PSSRU^3^ | Hospital-based doctors – consultant surgeon | |
| Registrar anaesthetist (per hour) | 55 | PSSRU^3^ | Hospital-based doctors – registrar | |
| Scrub nurse (per hour) | 64 | PSSRU^3^ | hospital-based nurses – grade 5/6 | |
| Circulating nurse (per hour) | 64 | PSSRU^3^ | hospital-based nurses – grade 2/3 | |
| Laparoscopic instrument pack (per operation) | 14 | HAREM hospitals’ pharmacy department | Laparoscopic appendicectomy only | |
| Laparoscopic ports (per operation) | 63 | HAREM hospitals’ pharmacy department | Laparoscopic appendicectomy only | |
| Extra laparoscopic graspers (per operation) | 32 | HAREM hospitals’ pharmacy department | Laparoscopic appendicectomy only | |
| Extra laparoscopic scissors (per operation) | 32 | HAREM hospitals’ pharmacy department | Laparoscopic appendicectomy only | |
| Ligating loop (per operation) | 16 | HAREM hospitals’ pharmacy department | Laparoscopic appendicectomy only | |
| Universal instrument pack (per operation) | 7 | HAREM hospitals’ pharmacy department | Open appendicectomy only | |
| Diathermy (per operation) | 17 | HAREM hospitals’ pharmacy department | Open appendicectomy only | |
| Sutures (per operation) | 2 | HAREM hospitals’ pharmacy department | Both open and laparoscopic appendicectomy | |
| Dressings (per operation) | 1 | HAREM hospitals’ pharmacy department | Both open and laparoscopic appendicectomy | |
| Anaesthetics (per operation) | 2 | BNF^4^ | Assuming amount required of 144mL for an average weight of male of 72 kg. | |
| **Antibiotic therapy in primary admission (per day)** | | | | |
| Co-amoxiclav (intravenously) | 3 | NHS trust pharmacy department and BNF^4^ | | Average dose of 1.2g every 8 hours. |
| Co-amoxiclav (oral therapy) | 1 | NHS trust pharmacy department and BNF^4^ | | Average dose of 500/125 mg 3 times a day. |
| Metronidazole (intravenously) | 1 | NHS trust pharmacy department and BNF^4^ | | Average dose of 500 mg every 8 hours |
| Metronidazole (oral therapy) | 1 | NHS trust pharmacy department and BNF^4^ | | Average dose of 400 mg 3 times a day. |
| Amoxicillin (intravenously) | 3 | NHS trust pharmacy department and BNF^4^ | | Average dose of 1g every 8 hours. |
| Amoxicillin (oral therapy) | 1 | NHS trust pharmacy department and BNF^4^ | | Average dose of 500 mg every 8 hours. |
| Cefuroxime (intravenously) | 6 | NHS trust pharmacy department and BNF^4^ | | Average dose of 500mg twice daily. |
| Cefuroxime (oral therapy) | 6 | NHS trust pharmacy department and BNF^4^ | | Average dose of 1.5g three times daily. |
| Gentamicin (intravenously) | 10 | NHS trust pharmacy department and BNF^4^ | | Average dose of 5 mg/kg subsequent doses adjusted according to serum-gentamicin concentration, to be given in a once daily dose regimen. |
| Tazocin (piperacillin with tazobactam; intravenously) | 16 | NHS trust pharmacy department and BNF^4^ | | Average dose 4.5g three times daily. |
| **Inpatient costs (per day)** | | | | |
| General ward | 435 | NHS Reference Costs^1^ | | NHS Reference Costs 2018/19 – Non-elective short stay. FD05B Abdominal Pain without interventions. |
| HDU bed | 967 | NHS Reference Costs^1^ | | NHS Reference Costs 2018/2019 – Critical care: XC07Z Surgical Adult Critical Care, 0 Organs Supported. |
| ICU bed | 1,614 | NHS Reference Costs^1^ | | NHS Reference costs 2018/2019 – Critical Care: Weighted average of currency codes XC01Z-XC06Z Adult Critical Care, 1-6 or more Organs Supported. |
| **Readmissions** | | | | |
| *Readmissions not involving surgery (per day)* | | | | |
| Day hospital stay | 407 | NHS Reference Costs^1^ | | NHS Reference Costs 2018/19 – Day case: FD05B Abdominal Pain without interventions. |
| Overnight hospital stay | 435 | NHS Reference Costs^1^ | | NHS Reference Costs 2018/19 – Non-elective short stay: FD05B Abdominal Pain without interventions |
| *Readmissions involving surgery (per patient)* | | | | |
| Appendicectomy | 2,922 | NHS Reference Costs^1^ | | NHS Reference Costs 2018/19 – Non-elective short stay: weighted average of day case FF37A-FF37E currency codes. |
| Right hemicolectomy and other proximal colon procedures | 4,421 | NHS Reference Costs^1^ | | NHS Reference Costs 2018/19 – Non-elective short stay: weighted average of day case FF32A-FF32C currency codes |
| Other major and complex general abdominal procedures | 2,421 | NHS Reference Costs^1^ | | NHS Reference Costs 2018/19 – Non-elective short stay. Weighted average of FF50A-FF50C and FF51A-FF61C currency codes. |
| Other intermediate and minor abdominal procedures | 934 | NHS Reference Costs^1^ | | NHS Reference Costs 2018/19 – Non-elective short stay. Weighted average of FF53A-FF53B currency codes. |

*BNF: British National Formulary, CT: Computed Tomography, HDU: High Dependency Unit, ICU: Intensive Care Unit, MRI: Magnetic Resonance Imaging, National Health Service, USS: Ultrasound Scan.

**Supplementary Table 4: Alternative cost assumptions for sensitivity analyses**

|  | **Base case** | **Sensitivity analysis** |
| --- | --- | --- |
| Overheads | Mean value across 2019 ISD Scotland trusts for gastroenterology | Considering 1st and 3rd quartile for overhead costs |
| Stump closure technique | Based on indicative cost of laparoscopic from HAREM hospitals’ pharmacy department | Considering costs for alternative appendiceal stump closure techniques |
| Proportion of laparoscopic procedures | Considering the proportion of laparoscopic surgeries observed in COVID:HAREM in the primary admission | Increasing the proportion of laparoscopic procedures up to 95% |
| Theatre staff | Two surgeons (consultant and registrar), two anaesthetists (consultant and registrar) and five nurses (scrub and circulating) | Considering 3 and 7 nurses in operating room |
| Operations in readmissions | 50% patients with missing data on operations in readmissions were assumed to receive major abdominal procedures. The rest were assumed to received minor/intermediate procedures. | Considering higher (75%) and lower (25%) proportion of major/complex procedures |
| Baseline covariates | Unadjusted analysis | Adjusted for age, sex, Rockwood clinical frailty index, BMI, use of diabetes medications, COPD, smoking history, MI history, use of immunosuppressive medications, active cancer and dementia |
| Distribution of costs | Normal | Gamma |

**Supplementary Table 5: Resource use up to 90 days by group**

|  | **Conservative management group (N=1,222)** | **Operative management group (N=1,222)** |
| --- | --- | --- |
| **Index admission resource use categories** | | |
| **Diagnostic imaging** | | |
| Diagnostic USS: n (%) | 258 (21) | 200 (16) |
| Diagnostic MRI: n (%) | 10 (1) | 12 (1) |
| Diagnostic CT: n (%) | 868 (70) | 852 (70) |
| **Antibiotic therapy** | | |
| Antibiotic treatment (days): mean (SD) | 6.20 (4) | 3.63 (4) |
| **Operations** | | |
| Number of operations: n (%) | 182 (15) | 1,199 (98) |
| Time in theatre (hours): mean (SD) | 0.18 (0) | 1.15 (0) |
| Level 2 (HDU) post-operative care (bed-days): mean (SD) | 0.01 (0) | 0.03 (0) |
| Level 3 (ICU) post-operative care (bed-days): mean (SD) | 0.03 (0) | 0.03 (0) |
| **Hospital length of stay** | | |
| General ward bed-days: mean (SD) | 3.08 (3) | 3.46 (4) |
| **Readmission resource use categories** | | |
| **Number of readmissions: n (%)** | 447 (37) | 246 (20) |
| **Number of operations in readmissions: n (%)** | 116 (9) | 35 (3) |

*Mean calculations done using total number of patients in the group.
